# Dental Education

**Published:** 1896-01

**Authors:** W. T. Jackman

**Affiliations:** Cleveland, Ohio.


### Dental Education.

BY W. T. JACKMAN D.D.S., CLEVELAND, OHIO.

Read before the Ohio State Dental Society, December 4th, 1895.

Mr. President and Members of the Ohio Dental Society: When the worthy President of this Society, Dr. Todd, requested me at the Tri-State meeting in Detroit, to write a paper for this meeting, I felt like reminding him of the scriptural injunction, "In honor preferring one another," and thus asking him to give the honor to the other fellow. But his persistence led me to conclude that possibly a duty toward this Society should be discharged, and this is the reason for acquiescing in his desire.

Dental education, like almost all other dental subjects, has been so thoroughly discussed of late that one is led to conclude there is hardly a division of this broad subject which has not been written or talked about. However, I believe we all concede that but a start has been made toward a final solution of certain phases of this, at the present time, all important subject.

When, where and how shall the dental student be trained for this most important of all secular callings?

What would be the best dental legislation for each State, or rather, how shall the legislation of the various States be made to harmonize?

What should be the literary attainments of the applicant for dental honors?

The answers to these and many kindred interrogatories have not, as yet, in the main, been satisfactory. The writer is glad to know, however, that these questions are being given much thought by the profession at present, and it is reasonable to conclude that the most of them will reach a satisfactory solution at no very distant day. If each writer on this subject can but add a single candle power of light to assist in illuminating the way, he will have done something toward solving these vexatious problems. If you will kindly give your attention for a few moments the writer will give you a few conclusions, in brief, pertaining to this many-sided subject. What shall be the literary attainments of the applicant for dental honors? Since this, of all others, is conceded to be the age of progress in the educational world, and if we desire, and we certainly do, to be known as a learned profession, or, if you prefer, a learned specialty in the medical profession, then the time has come to require a higher literary training; certainly nothing less than the evidence of graduation from high school, the possession of a teacher's certificate from the County Board of Examiners, or their equivalents for admission to our dental schools. Bishop Vincent said recently to a class of young men for clerical honors: "God does not so much call men to preach as He calls them to *prepare* to preach." So with dentistry. The crying need of the people to-day is for men and women to *prepare* to practice dentistry. If the applicant possesses the above literary training, then he should seek a tutorage, for at least one year, with some good dentist, not so much to actually *do* in this brief time as to see *how* to do. Let him spend the greater part of this year reading on operative and prosthetic technics and then he will be ready to take up his college work and push it with such a vim to a successful termination as the untutored cannot dream of. If he can afford to spend two years with a good preceptor, so much the better. Then follows his college course which should not be completed before twenty-one years of age. He should now be ready or prepared to begin the practice of dentistry.

It is, indeed, pleasing to note the advance the dental schools have made during the last decade, probably a greater advance in thorough practical teaching than in all their history before. In fairness to the schools organized in the last few years, it must be conceeded that they gave the impetus to nearly all this forward move for greater things. Whence came that modern idea, that idea which has almost revolutionized dental teaching—dental technics? Surely not from the old schools. Let it be understood we would not wrest a vestage of honor from the older schools, for they have done a splendid work; but it must be admitted that they have fallen into somwhat of a rut in their methods of teachings. When the National School of Dental Technics was organized, a year ago last August, at Old Point Comfort, it was gratifying to see those schools eagerly becoming members, and those who did not join at that time fell into line, we believe, this year at Asbury Park. So all are now on the same high plane, each eager to do its best for those who apply for instructions within its portals. The National Association of Dental Faculties has had much to do in bringing about these much-to be-desired results. While it is very pleasing to note the forward move by the colleges, it is with regret we are compelled to take cognizance of the fact that there is another body endowed with vast power which does not seem awake to its possibilities—we refer to the National Association of Dental Examiners. *It is to this body that the profession must look to harmonize the dental laws of the various States, and see to it that those States which have no dental law get one as soon as possible.*

We know it may be argued that the function of an examining board is to carry out certain provisions of law already made. This is true as applied to individual States, but not so, we take it, as applied to the National Association of Dental Examiners; for there is no national law to govern them, but rather their function is as stated above. We note but fourteen States and the District of Columbia were represented at the last meeting. This leads us to think that unless a better organization is effected much help along educational lines cannot be hoped for from this body by the profession—certainly not in the direction of dental legislation.

Let the National Association of Dental Examiners effect a *thorough* organization. It is suggested that a special effort be put forth to have every State in the Union, as well as every reputable school represented, no difference whether said State has a dental law or not. Get a representative dentist from that State to come, and try to get him enthused with the idea of the necessity of getting a dental law in his respective State, and help him to get it; for, as stated, this body should be the central authority from which all suggestions pertaining to dental legislation should come. This being true, then when this body deems it wise that dental laws should be made so and so, should be amended or revised, then the dentists of the various States should see that it be so. 'Tis true, gentlemen, to accomplish this will require a few years of hard, persistent work, but will not the results be worth infinitely more than the cost? Then will we have reached the point where one State cannot say to the practitioners of a sister State, even though they be graduates: "Our standard is higher than yours, therefore, if you wish to practice within our borders you must pass a special examination."

After a thorough organization of this body the first thing to be accomplished, in the present chaotic condition of legislation, is to see that every certificate gotten by actual examination from one State board is received by every other State board as *prima facie* evidence of the owner's right to practice dentistry. Volumes have been written on this point, but it seems to the writer this question can be settled very easily and with justice to all.

National legislation, barring the question of constitutionality, might be, after getting it, very unsatisfactory to the profession.

This national body, when organized as above, should, at each yearly meeting, formulate a list of questions and decide as to the nature of the clinics the applicant should give, for I do not believe a single person should be granted a certificate without his first having demonstrated his ability to *do* as well as having given satisfactory evidence of his *theoretical* knowledge. Then each board should use this list for the following year, the national body formulating a new list each succeeding year. This seems to be a solution of this matter " in a nut shell." In this way each State can ratify what the other does. Some may say that would necessitate a revision of some of the State laws. Grant that; would it not be an easy matter to convince legislators of the equity and justice of such a revision?

Just a word in regard to the formation of our State boards. The writer does not believe we shall ever have the kind of State boards we should have until their creation is divorced from politics. The State board should be the progeny of the State society. Its creation should be by election. Such men only should constitute our State boards who have a high conception of dental education—such men as will assist the colleges in their efforts to elevate the standard of dental education by making their test of the applicant's fitness even more severe than that of the colleges. If the National Association of Dental Examiners will create such a standard there will be no need of the States passing laws requiring that all applicants for certificates be graduates; for, if such a standard were created, then probably not more than one non-graduate would receive a certificate where a hundred now do.

Another plan is suggested for the formation of State boards and their duties pertaining to the harmonizing of legislation of the respective States, namely: Let each board be composed of not fewer than five members; when there are two or more dental schools in the State let there be two members for each school with an extra member, that the board be composed of an odd number; this board, then, should hold the final examination for the colleges within its State. When a student is given a diploma after such examination, said diploma should be a passport to practice dentistry in any State in the Union. This State body to be governed, of course, by the national body as outlined above. This would relieve the various dental faculties from the responsibility of the final examinations and thus whatever of odium might be attached to the granting of degrees to supposedly incompetent persons would be removed, at least, from the faculties.

The central thought of this paper, as you will have noticed, has been to suggest some ways, without entering much into detail, out of the legislative tangle the profession is now in. Whatever of merit or demerit these suggestions may have, it is hoped that this society will thoroughly discuss this feature of the paper and put itself on record so that eventually the profession may arrive at something definite pertaining to this phase of dental education, viz: dental legislation.

---

Don't use soap and water on the body just preceding the application of cocaine; the alkali destroys the anæsthetic action.

